# Increased Expression Levels of Metalloprotease, Tissue Inhibitor of Metalloprotease, Metallothionein, and p63 in Ectopic Endometrium: An Animal Experimental Study

**DOI:** 10.1055/s-0038-1675612

**Published:** 2018-11

**Authors:** Verônica Cristina Moraes Brandão, Juliana Meola, Sergio Britto Garcia, Francisco José Candido-dos-Reis, Omero Benedicto Poli-Neto, Antonio Alberto Nogueira, Julio Cesar Rosa-e-Silva

**Affiliations:** 1 Faculty of Medicine, Universidade de São Paulo, Ribeirão Preto, SP, Brazil; 2 Faculty of Medicine, Universidade Federal do Mato Grosso, Cuiaba, MT, Brazil

**Keywords:** endometriosis, cell differentiation, cell proliferation, tissue invasion, endometriose, diferenciação celular, proliferação celular, invasão tecidual

## Abstract

**Objective** To characterize the patterns of cell differentiation, proliferation, and tissue invasion in eutopic and ectopic endometrium of rabbits with induced endometriotic lesions via a well- known experimental model, 4 and 8 weeks after the endometrial implantation procedure.

**Methods** Twenty-nine female New Zealand rabbits underwent laparotomy for endometriosis induction through the resection of one uterine horn, isolation of the endometrium, and fixation of tissue segment to the pelvic peritoneum. Two groups of animals (one with 14 animals, and the other with15) were sacrificed 4 and 8 weeks after endometriosis induction. The lesion was excised along with the opposite uterine horn for endometrial gland and stroma determination. Immunohistochemical reactions were performed in eutopic and ectopic endometrial tissues for analysis of the following markers: metalloprotease (MMP-9) and tissue inhibitor of metalloprotease (TIMP-2), which are involved in the invasive capacity of the endometrial tissue; and metallothionein (MT) and p63, which are involved in cell differentiation and proliferation.

**Results** The intensity of the immunostaining for MMP9, TIMP-2, MT, and p63 was higher in ectopic endometria than in eutopic endometria. However, when the ectopic lesions were compared at 4 and 8 weeks, no significant difference was observed, with the exception of the marker p63, which was more evident after 8 weeks of evolution of the ectopic endometrial tissue.

**Conclusion** Ectopic endometrial lesions seem to express greater power for cell differentiation and tissue invasion, compared with eutopic endometria, demonstrating a potentially invasive, progressive, and heterogeneous presentation of endometriosis.

## Abstract (pt)

**Resumo:**

**Objetivo** Caracterizar o padrão de diferenciação celular, proliferação e invasão tecidual em endométrio eutópico e ectópico de coelhas com lesões de endometriose induzidas por um modelo experimental 4 e 8 semanas após o procedimento de implantação endometrial.

**Métodos** Vinte e nove coelhas fêmeas Nova Zelândia foram submetidas a laparotomia para indução de endometriose através da ressecção de um dos cornos uterinos, isolamento do endométrio e fixação do tecido no peritônio pélvico. Dois grupos de animais (14 animais em um grupo e 15 animais no outro) foram sacrificados 4 e 8 semanas após a indução da endometriose. A lesão foi excisada junto com o corno uterino contralateral para determinação da presença de glândulas e de estroma endometrial. Reações de imunohistoquímica foram realizadas no tecido endometrial eutópico e ectópico para análise dos seguintes marcadores: metaloprotease (MMP9) e inibidor tecidual da metaloprotease 2 (TIMP-2), os quais estão envolvidos na capacidade de invasão do tecido endometrial; e metalotioneina (MT) e p63, os quais estão envolvidos na diferenciação e proliferação celular.

**Resultados** A intensidade da imunomarcação para MMP9, TIMP-2, MT e p63 foi mais alta nos endométrios ectópicos do que nos endométrios eutópicos. Contudo, quando as lesões foram comparadas entre 4 e 8 semanas, nenhuma diferença foi observada, com exceção do marcador p63, o qual foi mais evidente depois de 8 semanas de evolução do tecido endometrial ectópico.

**Conclusão** Lesões endometriais ectópicas parecem expressar maior poder de diferenciação celular e de invasão tecidual comparadas com endométrios eutópicos, demonstrando o potencial de invasão, de progressão e de apresentação heterogênea da endometriose.

## Introduction

The etiopathogenesis of endometriosis is controversial.^1^ Several theories have been proposed, such as the presence of retrograde menstrual flow associated with an immunological predisposition in the peritoneal microenvironment that facilitates the implantation of viable endometrial cells and has the potential for implantation.^2^ Recent studies suggest that the endometria of patients with endometriosis have an invasive and aggressive behavior, and higher expression levels of substances related to cellular invasion, cellular differentiation, and proliferation, such as metalloproteases (MMPs) and tissue inhibitors of MMPs (TIMPs),^3^ ^4^ ^5^ ^6^ ^7^ ^8^ ^9^ ^10^ ^11^ p63,^12^ ^13^ ^14^ ^15^ ^16^ and metallothionein (MT)^17^ ^18^ ^19^ have been described.

Metalloproteases are a family of endopeptidases that play a role in degrading and remodeling the extracellular matrix. They are zinc dependent and include collagenase, gelatinase, and stromal enzymes. Their activities are regulated by TIMPs.^20^ The production of MMPs and of TIMPs occurs in the endometrial stroma and in the epithelium, as well as in polymorphonuclear leucocytes. Another important source of these enzymes are macrophages, neutrophils, and eosinophils, activated in response to a certain degree of inflammation present in the peritoneal cavity of women with endometriosis.^21^ ^22^ ^23^

The membrane protein p63 is a marker of cell differentiation, homologous to the tumor protein suppressor p53, and is expressed in basal squamous and subcolumnar reserve cells in the uterine cervix, in the breasts, in the salivary glands, and in the prostate.^24^ It regulates proliferation and epithelial differentiation.^25^

Metallothionein is a low-molecular-weight protein that performs functions in cell growth, repair, and proliferation.^26^ The perinuclear location of MT is known to be important in the protection against DNA damage and apoptosis induced by external stressors.^27^ ^28^

The use of female rabbits in experimental models of endometriosis is characterized by the development of homogeneous lesions, generally solid hemorrhagic masses, which are easily produced through an autotransplant of endometrial fragments or through the opening and exposure of the endometrial cavity.^29^ ^30^ In addition, rabbits were chosen as the experimental animals because of their low infection rate, which makes antibiotic administration unnecessary.^31^

In the experimental model conducted in our service,^32^ the development of lesions after endometrial tissue implantation was 100% after 4 and 8 weeks, with the presence of stroma and gland on histological observation, a fact also reported in other experimental studies.^31^ ^33^

The objective of the present study was to characterize the proliferation, differentiation, and invasion behavior in eutopic and ectopic endometria in rabbits submitted to the induction of endometriosis lesions by using a known experimental model, 4 and 8 weeks after the endometrial implantation procedure.

## Methods

### Animals

The present study was performed in the experimental surgery sector of the department of surgery and anatomy of the Hospital das Clínicas, Ribeirão Preto, State of São Paulo and in the department of pathology of the Faculty of Medicine of Riberão Preto of the Universidade de São Paulo, state of São Paulo, Brazil. It was approved by the ethics committee for animal experimentation by the same institution. After the sample calculation, the size of the study group was set at 10 animals. However, considering the possibility of losses, 15 adult animals were included per group, with one loss (death) during the experiment, therefore totaling 29 adult New Zealand female virgin rabbits from the vivarium of the Faculty of Medicine of Ribeirão Preto. The rabbits were kept in appropriate cages under the same conditions for 3 days before the induction of the lesions. All of the rabbits were submitted to a laparotomy under general anesthesia with intravenous administration of 3 mL of thionembutal (2.5%) along with 1 mL of xylestesin (2%).

### Induction Technique for Endometriotic Lesions

The pelvic cavity was opened with a median longitudinal incision of ∼ 2 cm in length, 2 cm from the pubis of the animal. Then, ∼ 4 cm of the right uterine horn was resected and then the horn was closed. The portion of the uterine that was resected was immersed in 0.9% saline solution for ∼ 2 minutes for tissue cleaning and then cut longitudinally, resecting a 5 × 5-mm fragment. This endometrial tissue fragment was sutured to the peritoneum near the reproductive tract of the rabbits by using 2 simple Vicryl 6.0 (Ethicon Inc., Sommerville, NJ, USA) sutures, with the endometrium was facing inward facing the abdominal cavity, with posterior closing of the abdominal surgical incision. No hormonal supplements were administered before or after the laparotomy. The same observer performed all of the procedures.^32^

### Removal of Lesions for Histological Analysis

The rabbits were divided into two groups, namely group 1, which consisted of 14 animals whose lesion evolution time was 4 weeks, and group 2, which consisted of 14 animals with a lesion evolution time of 8 weeks. After pelvic inspection, identification, and documentation of the lesion, the rabbits were sacrificed. The lesion, along with the left uterine horn (contralateral), was excised for histological analysis. The excised tissues were set in 10% formaldehyde and processed for inclusion in paraffin. After preparing the slides, the tissues were stained with hematoxylin and eosin (H&E) stain for histological analysis. The analysis results indicated that 100% of the samples in both groups were composed of active endometrial (gland and stroma) tissues, with similar morphological characteristics. The lesions were characterized by thin-walled cysts located on the striated muscle of the abdominal wall, projecting toward the abdominal cavity. The cyst walls were formed by a thin layer of connective tissue, rich in cells, and covered by simple squamous epithelium. The stroma is rich in fibroblasts, and contains some macrophages and eosinophils in addition to a large quantity of typical endometrial glands. The evaluation of the lesions in two stages has the purpose of verifying the presence of any tissue modification during the progression of the lesions, as well as their growth.

### Immunohistochemical Techniques

Histological sections (4–5 mm) were submitted for histochemical analysis with the antigen-antibody reaction. The reaction was developed by using a marker visible under the microscope. The deparaffinized and hydrated sections were recovered antigenically by incubation in a buffered medium in a steam pot for 40 minutes. After cooling, the endogenous tissue peroxidases were removed by adding hydrogen peroxide, and horse serum was added to prevent nonspecific binding of the primary antibody. The slides were then incubated with primary antibodies obtained from Novocastra Laboratories Ltd. (Newcastle upon Tyne, United Kingdom). The samples were then evaluated regarding the following markers: MT (1:100; clone E9; Dako North America Inc., Carpinteria, CA, USA) and p63 (1:500; clone BC4A4; Biocare Medical, Concord, CA, USA), which are related to cell proliferation and differentiation, and MMP 9 (1:100 clone 15W2; Leica Biosystems, Wetzlar, Germany) and TIMP-2 (1:100 clone 3A4; Novocastra Laboratories, United Kingdom), which are involved with invasive capacity. The materials were then incubated with the secondary antibody and submitted to the avidin-biotin step. The reaction was developed by treatment with 3.30-diaminobenzidine (Sigma-Aldrich Inc., St. Louis, MO, USA) for 5 minutes. Thereafter, the materials were counterstained with Harris H&E stain and mounted on slides. The immunohistochemical markers were analyzed quantitatively and manually, counting the number of marked cells in thousands, and divided into 4 quadrants of 250 cells each. All of the slides were evaluated by two pathologists experienced in immunohistochemistry who were blinded to the type of tissue to be analyzed.

### Statistical Analysis

A statistical analysis was performed by using the GraphPad Prism 5.0 32-bit executable software (GraphPad Software Inc., San Diego, CA, USA). The paired Student *t*-test was used for variables with a normal distribution and for comparison of the paired data. The non-paired Student *t*-test was used for variables with normal distribution and non-paired data. Finally, the Mann-Whitney U-test was used for non-paired variables with non-normal distribution. The level of statistical significance was set at 5%.

## Results

**Fig. 1:**
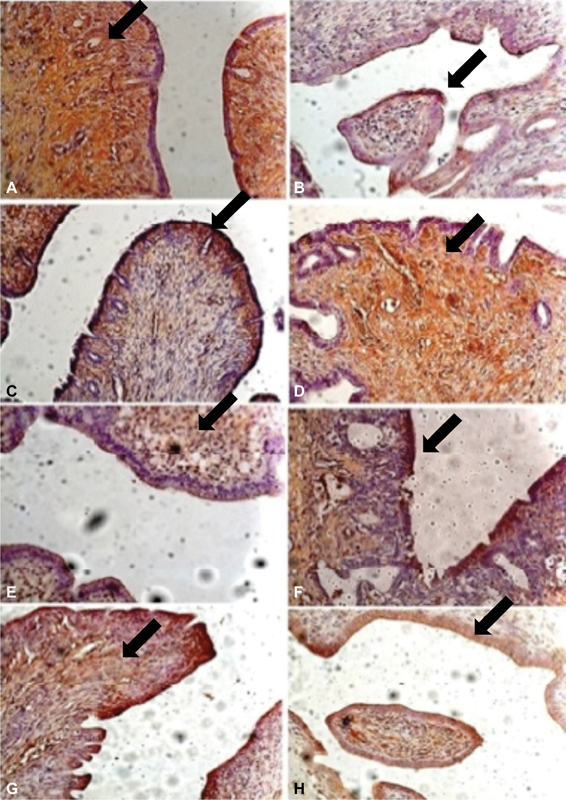
Immunostaining by: metalloprotease in the eutopic (A) and ectopic endometria (B); tissue inhibitor of metalloprotease in the eutopic (C) and ectopic endometria (D); metallothionein in the eutopic (E) and ectopic endometria (F); and p63 in the eutopic (G) and ectopic endometria (H). 40x enlargement.

A significantly more intense stain for MMP9, TIMP-2, MT, and p63 were observed in the ectopic endometria than in the eutopic endometria. (Table 1 and Fig. 1). However, regarding the ectopic lesions that were compared at 4 and 8 weeks, no significant differences were observed, with the exception of the p63 indicator, which was more evident after 8 weeks of ectopic endometrial tissue progression. (Table 2 and Fig. 1).

**Table 1:** Cellular immunostaining according to endometrial types

	Topic mean* ± *SD	Ectopic mean* ± *SD	*p*-value
MMP-9	0.273 ± 0.147	0.364 ± 0.223	0.0003
TIMP-2	0.249 ± 0.126	0.274 ± 0.152	0.02
Metallothionein	0.277 ± 0.141	0.361 ± 0.220	0.0003
p63	0.218 ± 0.080	0.276 ± 0.095	< 0.0001

Abbreviations: MMP-9, metalloprotease; SD, standard deviation; TIMP-2, tissue inhibitor of metalloprotease.

**Table 2:** Cellular immunostaining in ectopic endometrium according to progression time

	4 weeks mean* ± *SD	8 weeks mean* ± *SD	*p*-value
MMP-9	0.418 ± 0.209	0.314 ± 0.231	0.21
TIMP-2	0.288 ± 0.152	0.262 ± 0.157	0.66
Metallothionein	0.428 ± 0.221	0.298 ± 0.208	0.11
p63	0.240 ± 0.058	0.309 ± 0.113	0.05

Abbreviations: MMP-9, metalloprotease; SD, standard deviation; TIMP-2, tissue inhibitor of metalloprotease.

## Discussion

In the present study, we have demonstrated that the ectopic endometrial lesions showed a higher number of immune stained cells expressing cellular invasion markers (MMP-9 and its inhibitor TIMP-2) and of molecules involved in cellular proliferation and differentiation (MT and p63) than the eutopic endometrial lesions, using a rabbit experimental model of endometriosis. However, when 4- and 8-week ectopic lesions were compared, there was no significant difference except for the p63 marker, which was more evident after 8 weeks of evolution of the ectopic endometrial tissue. This suggests that this marker increases the longer the period of lesion evolution, demonstrating a greater capacity of differentiation of the ectopic endometriotic foci.^34^

The MMP system is formed by an enzymatic component, MMP, and by an inhibiting component, the TIMPs. It has been well established that this system performs a critical role during the normal development and growth of the endometrium, as well as many other physiological processes.^35^

Metalloproteases have been implicated in the pathogenesis of endometriosis, the greater part of which are synthesized during the proliferative phase and stimulated by estrogen expression. Inversely, progesterone levels reduce MMP transcription and secretion. In an endometriosis model using mice and human endometria, endometrial treatment with estrogen led to an increase in MMP production and in endometrial ectopic implantation. In contrast, treatment with progesterone, which inhibits the production of MMP^36^ or TIMP-1, decreased the success rate of ectopic implantation.^37^ ^38^ ^39^

Metalloproteases work on the dynamics of the degradation and remodeling process of the extracellular matrix, stimulating cellular proliferation and apoptosis, as well as inducing cellular migration. Several previous studies suggest that eutopic and ectopic endometria in women with endometriosis present altered levels of the MMP/TIMP system, indicating that these two enzymes play a role in the pathogenesis of the illness. In general, the irregular synthesis and secretion of MMPs by endometriosis lesions, combined with aberrant quantities of TIMP-1 in the peritoneal fluid, could disturb the normal proteolytic environment of the peritoneal cavity, thus inducing a more aggressive behavior and facilitating invasion by ectopic cells. The exact mechanisms that lead to the aberrant expressions of MMPs and their tissue inhibitors in endometriosis have yet to be defined.^3^ ^36^ ^40^ ^41^ ^42^ ^43^ ^44^ ^45^ ^46^ ^47^

Meanwhile, studies inferred that the increased expression levels of MMPs in the peritoneal fluid and in ectopic lesions in endometriosis patients could be a secondary event that results from an innate difference in peritoneal and systemic factors instead of from alterations in the endometrium, causing an abnormal peritoneal response to the menstrual reflux, which in turn facilitates ectopic implantation.^48^

Vinatier et al (2000)^49^ suggested that the characteristics of endometrial cells differ between women with and without endometriosis. The endometrium in women with endometriosis has an increased capacity for proliferation and implantation in ectopic locations. Therefore, the interpretation of the results should consider that eutopic endometrial cells and refluxed cells in the peritoneal cavity are not regulated by the same environment. Refluxed cells in the peritoneal cavity are regulated by the microenvironment of the peritoneal fluid, contrary to eutopic endometrial cells, which are regulated by blood flow factors. This concept of a different environment is rarely considered when eutopic and ectopic endometria are compared. The differences that are observed should be interpreted not only as differences in tissue characteristics, but also as a result of environmental differences. The type of cellular modification along with local factors such as the microenvironment of the peritoneal fluid or the intraovarian environment will determine if they will develop typical lesions, deep endometriosis, or ovarian cysts.^49^ ^50^

The present study observed a higher immunostaining intensity of MMP-9 and its inhibitor, TIMP-2, in ectopic endometria, which was related to a higher ability to proliferate and to the invasive capacity of these tissues, as shown in previous studies.^3^ ^4^ ^5^ ^6^ ^7^ ^8^ ^9^ ^10^ ^11^

Although the progression time of these lesions did not interfere in a statistically significant way in the MMP-9 (*p* = 0.214) and in the TIMP-2 staining (*p* = 0.66), our results suggest that the evaluation of MMP-9 and TIMP-2 could be used as a prognostic indicator of endometrial invasion; therefore, increasing the proteolytic activity would be one of the many factors that contribute to the invasive properties of the endometrium, resulting in the development of endometriosis.

The molecular alterations observed in the human endometrium during the menstrual cycle could be crucial to the reproductive function. The accumulation of cytotoxic cells increases the exposure of endometrial cells to apoptosis, and protection against this process could be reached by self-regulating cellular mechanisms, suggesting that MT participates in this context. Significant differences in MT expression observed in the endometrium regarding alterations in the menstrual cycle could suggest the participation of MT in the protection against apoptosis in endometrial cells.^51^

Metalloprotease takes part in the detoxification process of organisms, being found in benign and malignant neoplasms, among others, in animals and humans, mainly in the S phase of the cellular cycle. It is considered as an index for cell proliferation and tumor progression. During proliferation, epithelial cells have a higher expression level of MT, indicating an increase in the number of dividing cells, particularly during the S phase of the cell cycle. This is why MT could be considered as a marker of endometriosis.^18^

The perinuclear location of MT is known to be important for protection against DNA damage and apoptosis induced by external stressors.^27^ Therefore, the MT expression in endometrial cells could favor their persistence in ectopic localization, as reported by Wicherek et al (2006).^19^

We have observed a higher-intensity stain in ectopic endometrial tissue in comparison with eutopic tissue; however, the difference was not statistically significant regarding the progression time of the lesion in the ectopic tissue.

Data suggest that MT expression seems to be under hormonal control in normal endometrium and that MT could modify the p53 expression and be used as a biological marker of aggressive behavior in endometrial lesions.^17^ The ability of the endometrium to distinguish cytotoxic activity from increased protection against DNA damage (MT expression), as well as concomitant changes in the number of cells in the immune system and its activity, which are observed in normal endometrium during the phases of the menstrual cycle, seems to be fundamental for the pathological characteristics of endometriosis.^52^

The p63 protein has been described as a marker of basal and reserve cells in the female genital tract,^53^ ^54^ ^55^ being strongly related with altered differentiation, including metaplasia, either isolated or in combination with neoplasms.^56^ ^57^ Some clinical and laboratory data provide evidence that suggests that ectopic endometrial lesions result in the dislocation of basal endometrial cells.^58^

Studies showed that endometriotic lesions express p63 differently; however, whether the lack of p63 expression in some lesions is related to the extent of the illness, to its clinical behavior, or to the exacerbation of the symptoms that accompany it is unclear.^13^

We have noted in our experimental model that the stain was more evident in ectopic endometria than in eutopic endometria (*p* < 0.0001), inferring a greater potential for differentiation in eutopic tissue, favoring the establishment of endometriosis, as it has already been suggested that p63-positive cells in normal endometria represent cells with a stem cell phenotype that have the potential for multidirectional differentiation.^12^ ^13^ ^14^ ^15^ ^16^

Another relevant fact was the interference of the progression time of the lesion regarding the staining intensity, which could suggest that the longer the progression, the greater the ability of endometriotic foci in the ectopic tissue to differentiate.

## Conclusion

Upon analyzing the different markers of cell proliferation and differentiation, as well as of tissue invasion in eutopic and ectopic endometria in the rabbits submitted to the induction of endometriotic lesions by the experimental model, 4 and 8 weeks after the endometrial implantation procedure, we conclude that the ectopic lesions seem to express a greater ability for cell proliferation and differentiation, as well as for tissue invasion when compared with eutopic endometria. This is evident in the greater intensity of the immunostaining for the proteins involved in the capacity to invade tissues (MMP-9 and TIMP-2) in ectopic endometria compared to the eutopic endometrium and in the higher number of cells of molecules involved in cell proliferation and differentiation (MT and p63) that were stained in ectopic endometria, which was most evident by the p63 stain in the endometrium after 8 weeks of progression. The ectopic endometrial lesions seem to express a greater ability for cell differentiation and tissue invasion than eutopic endometrial lesions, characterizing endometriosis as a potentially invasive, progressive, and heterogeneous disease in its presentation. However, more studies are necessary to better clarify the participation of these markers in the complex pathophysiological mechanism of endometriosis.
